# The dynamic interplay between cardiac mitochondrial health and myocardial structural remodeling in metabolic heart disease, aging, and heart failure

**DOI:** 10.20517/jca.2022.42

**Published:** 2023-01-03

**Authors:** Benjamin Werbner, Omid Mohammad Tavakoli-Rouzbehani, Amir Nima Fatahian, Sihem Boudina

**Affiliations:** Department of Nutrition and Integrative Physiology, University of Utah, Salt Lake City, UT 84112, USA.

**Keywords:** Mitochondria, mitophagy, fibrosis, fibroblasts, remodeling, cardiac

## Abstract

This review provides a holistic perspective on the bi-directional relationship between cardiac mitochondrial dysfunction and myocardial structural remodeling in the context of metabolic heart disease, natural cardiac aging, and heart failure. First, a review of the physiologic and molecular drivers of cardiac mitochondrial dysfunction across a range of increasingly prevalent conditions such as metabolic syndrome and cardiac aging is presented, followed by a general review of the mechanisms of mitochondrial quality control (QC) in the heart. Several important mechanisms by which cardiac mitochondrial dysfunction triggers or contributes to structural remodeling of the heart are discussed: accumulated metabolic byproducts, oxidative damage, impaired mitochondrial QC, and mitochondrial-mediated cell death identified as substantial mechanistic contributors to cardiac structural remodeling such as hypertrophy and myocardial fibrosis. Subsequently, the less studied but nevertheless important reverse relationship is explored: the mechanisms by which cardiac structural remodeling feeds back to further alter mitochondrial bioenergetic function. We then provide a condensed pathogenesis of several increasingly important clinical conditions in which these relationships are central: diabetic cardiomyopathy, age-associated declines in cardiac function, and the progression to heart failure, with or without preserved ejection fraction. Finally, we identify promising therapeutic opportunities targeting mitochondrial function in these conditions.

## Graphical Abstract

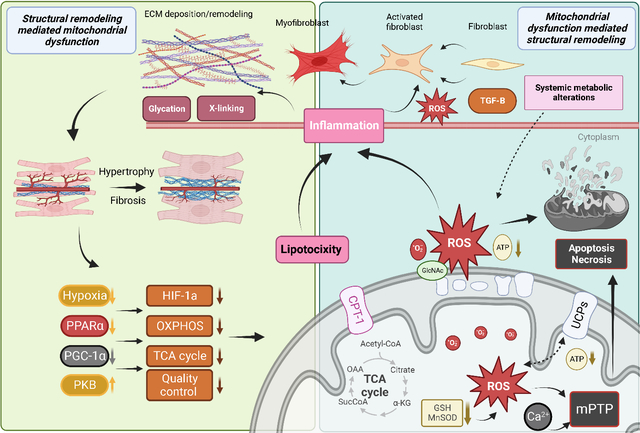

## TRIGGERS OF CARDIAC MITOCHONDRIAL DYSFUNCTION

### Systemic metabolic alterations

Based on recent National Health and Nutrition Examination Survey data, 35% of US adults and 55% of US adults over 60 years old meet the criteria for metabolic syndrome^[1]^. Systemic metabolic alterations such as hyperlipidemia and hyperglycemia are hallmarks of the suite of symptoms associated with this increasingly prevalent syndrome and constitute a primary risk factor for cardiovascular disease and mortality^[2]^. In addition to their deleterious effects on the micro- and macro-vasculature, such metabolic defects can also precipitate detrimental structural and functional alterations in the heart, including direct and indirect effects on cardiac mitochondrial bioenergetics, leading to cardiomyopathies and heart failure^[3]^. These include increased mitochondrial oxidative stress and impaired mitochondrial calcium handling, leading to mitochondrial dysfunction, and eventually cardiomyocyte cell death.

The metabolically ill myocardium has long been characterized by an increased reliance on free fatty acids (FFAs) and reduced glucose oxidation^[4]^. It has been proposed that this increased reliance may initially arise from elevated circulating FFA levels mediated by insulin resistance. Hyperphagic mouse models of diabetes (ob/ob and db/db) show increased FFA oxidation in the heart, increased myocardial oxygen consumption, and decreased cardiac contractile function^[5,6]^. These observations are consistent with those from human diabetic hearts, where insulin resistance and obesity were correlated with an increase in myocardial oxygen consumption and impaired cardiac function^[7]^. In addition, glucose utilization might be diminished due to insulin resistance, impaired pyruvate dehydrogenase activity, and decreased glucose transporter type 4 (GLUT4) content. Additionally, increased levels of FFA have been shown to reduce glucose metabolism through dysregulation of insulin receptor signaling^[1,8]^.

**Figure 1.**
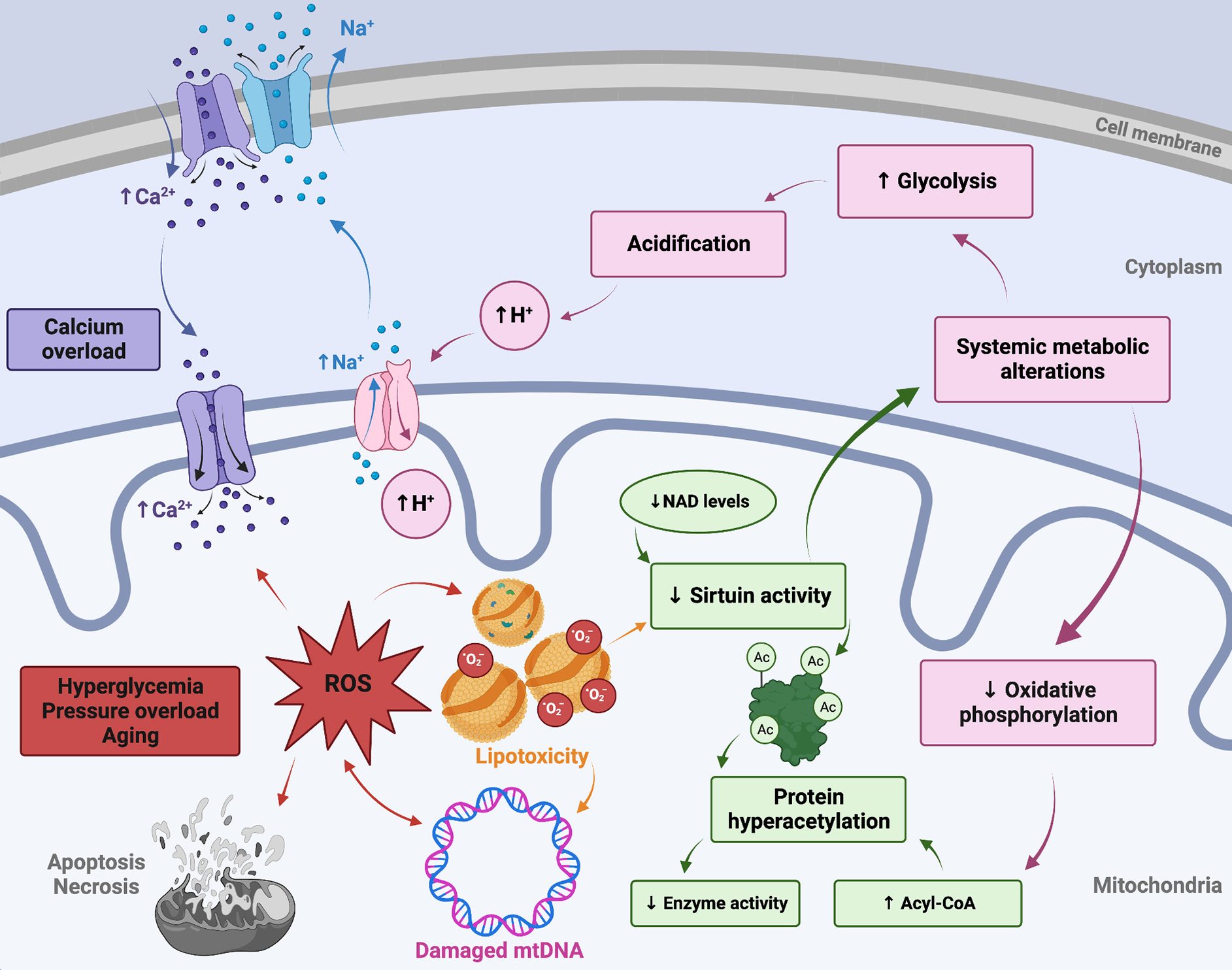
Altered systemic metabolism, aging, protein hyperacetylation, and calcium overload all mechanistically contribute to cardiac mitochondrial dysfunction. ROS: Reactive oxygen species; mtDNA: mitochondrial DNA. See Sections “Systemic metabolic alterations” and “Cardiac aging” for further details.

Reactive oxygen species (ROS) are free-radical and oxidant products derived from the one-electron reduction of molecular oxygen, including superoxide, hydrogen peroxide, and hydroxyl radicals, which are involved in both normal and pathological cell-signaling cascades. Superoxide radical is produced intracellularly within the mitochondrial electron transport chain of all cells, as well as by xanthine oxidase and the membrane-bound nicotinamide adenine dinucleotide phosphate (NADP)H-oxidase. Both enzymatic (superoxide dismutase (SOD), catalase, glutathione peroxide, *etc.* ) and non-enzymatic (vitamins E, C, ubiquinone, *etc.* ) antioxidant systems serve to maintain dynamic redox balance, preserving low concentrations of ROS involved in physiologic signaling pathways while mitigating pathological oxidative damage^[9]^. An imbalance in the production of ROS and free-radical scavenging antioxidant defense systems results in biochemical damage to crucial cellular macromolecules such as DNA, proteins, and lipids via oxidation [Figure 1]^[10]^. In addition to increased ROS production, many heart failure phenotypes exhibit downregulated ROS scavenging systems^[10,11]^. It is important to note that increased mitochondrial oxidative stress during the onset of heart failure has been recognized as both a cause and a consequence of the cascade of mitochondrial dysfunction and cardiac structural remodeling^[12]^; this bi-directional interplay is the focus of the current work and will be discussed in further detail throughout.

In the context of systemic metabolic alterations, it has been shown that cardiac insulin receptor knockout mice exhibit reduced glucose uptake and increased ROS generation, promoting mitochondrial dysfunction^[13,14]^. Previous work from our lab has shown that loss of insulin signaling may potentiate mitochondrial uncoupling and lead to increased ROS production, further impairing mitochondrial bioenergetics^[15]^. Accumulation of mitochondrial-derived ROS also plays a pivotal role in cardiomyocyte dysfunction, as will be discussed in detail subsequently, and it has been shown that hyperglycemia-induced ROS ultimately triggers the mitochondrially-mediated apoptosis pathway^[16,17]^.

Another mechanism by which systemic metabolic alterations may trigger mitochondrial dysfunction is through impairment of mitochondrial calcium handling. Several important mitochondrial metabolic enzymes are regulated by calcium, including pyruvate dehydrogenase, isocitrate dehydrogenase, a-ketoglutarate dehydrogenase, and ATP synthase (mitochondrial complex V). Calcium is also involved in crucial mitochondrial regulatory processes, and dysregulated calcium homeostasis is a hallmark of heart disease. Hyperglycemia, insulin resistance, and hyperinsulinemia have all been associated with calcium overload^[18]^ and studies have demonstrated impaired calcium handling and accumulation in diabetic animal models^[19,20]^. While the exact etiology of altered mitochondrial calcium handling is not yet fully elucidated, the mitochondrial calcium uniporter has long been recognized to play a central role in the microdomain localization of intracellular calcium, driving both mitochondrial energy metabolism and cell-death pathways in a concentration-dependent manner. Further elucidation of the macromolecular structure of the mitochondrial calcium uniporter complex has allowed for the use of additional genetic models to study the causes and consequences of uniporter calcium handling and altered calcium homeostasis *in vivo*, as reviewed elsewhere^[21–24]^. Additionally, it is known that sarcoplasmic reticulum calcium release results in increased cytosolic calcium concentrations leading to calmodulin-dependent depolarization of the mitochondrial membrane potential and opening of the mitochondrial permeability transition pore (mPTP), exacerbating mitochondrial dysfunction, and inducing cell death^[25,26]^. Additionally, a shift from oxidative phosphorylation (OXPHOS) to glycolysis in the metabolically dysregulated heart can result in cellular acidification, activating the Na^+^/H^+^ ion exchanger, leading to the accumulation of Na^+^ in the cytoplasm. This results in inverted Na^+^/Ca^2+^ ion exchanger activity, leading to intracellular and mitochondrial calcium overload [Figure 1]^[27]^. On the other hand, decreased intramitochondrial calcium in cardiomyocytes exposed to hyperglycemia has also been reported^[28,29]^.

Dyslipidemia is strongly and consistently implicated in the pathogenesis of atherosclerotic cardiovascular disease and cardiovascular mortality. In particular, elevated low-density lipoprotein (LDL) and diminished high-density lipoprotein (HDL) levels have proved to be consistent epidemiologic markers of cardiovascular mortality risk. While most studies have focused primarily on vascular function in this context, it is becoming increasingly recognized that myocardial metabolism, energetics, and whole-heart function are also impaired with dyslipidemia^[30,31]^. In fact, it has been suggested that HDL-associated molecules play a crucial role in maintaining cardiac mitochondrial function. For example, the HDL-related antioxidant enzyme Paraoxonase 1 has been shown to prevent damage to mitochondrial respiratory complexes by reducing oxidized lipid content^[32]^, while apolipoprotein A-1 has been shown to stabilize electron transport chain complex II and inhibit oxidative damage to the respiratory complexes via interactions with Coenzyme Q^[33]^. Additionally, concomitant knockout of the LDL receptor and apolipoprotein E-1 in mice showed enhanced tricarboxylic acid (TCA) cycle function and mitochondrial biogenesis, leading to improved cardiac mitochondrial function^[34]^, while a single knockout of the LDL receptor was associated with increased cardiac mitochondrial ROS production and a disturbance in mitochondrial membrane permeability^[35]^. Studies of apolipoprotein E-1 knockout mice reveal that hypercholesterolemia results in significant damage to cardiac mitochondrial DNA (mtDNA)^[36]^.

Insulin resistance, hyperglycemia, and dyslipidemia can lead to lipotoxicity, which is the deleterious effects of lipid accumulation in non-adipose tissues, which may be caused by an accumulation of ROS, ceramides, and acylcarnitines^[37]^. Increased levels of acylcarnitines have been associated with mitochondrial-mediated incomplete beta oxidation^[38]^. On the other hand, mitochondrial uncoupling and futile cycling may serve as important adaptive mechanisms to protect the heart from damage caused by lipotoxicity. Uncoupling proteins (UCPs) function as FA anion exporters by switching fatty acids out of the mitochondrial matrix, lowering the proton gradient, and diminishing oxidative burden^[39]^. Studies have also indicated that enhanced peroxisome proliferator-activated receptor (PPAR) expression can reduce incomplete FA oxidation and regulate transcription of FA metabolism^[40]^. Therefore, dysregulation in any of the mentioned pathways can lead to lipid abnormalities and mitochondrial dysfunction.

Cardiac hypertrophy typically develops as an adaptive response to maintain cardiac output when challenged with an increased workload^[41]^, and to a lesser extent, in response to growth factors and genetic mutations. Conditions of aortic stenosis, aortic constriction, and arterial hypertension are all examples of pressure overload (PO) that result in concentric cardiac hypertrophy^[42–44]^. Various animal models have been developed to study the causes and effects of cardiac hypertrophy, which often share a common theme of investigating alterations in mitochondrial structure or function during and after hypertrophic remodeling^[45,46]^. The metabolic alterations most frequently associated with hypertrophy tend to revolve around changes to fatty acid oxidation (FAO) and glucose utilization, but specific trends are not monotonic during the remodeling process and will be discussed in further detail in subsequent sections.

Briefly, many studies have described impaired myocardial FAO during PO induced cardiac hypertrophy and heart failure^[47,48]^. Recent studies suggest that PO may cause the accumulation of myocardial triglycerides (TGs), an indication of lipotoxicity^[49]^. In addition, neurohormonal changes, such as increased adrenergic activity, enhance adipose tissue lipolysis lead to increased delivery of FFAs to the heart^[50,51]^. Concomitant with defective FA utilization, increased FFA delivery promotes fat accumulation and lipotoxicity in cardiomyocytes^[52,53]^. On the other hand, studies have shown an increase in glycolysis in PO induced cardiac hypertrophy, without an increase in glucose oxidation^[54]^. Such a shift toward glycolytic metabolism in the heart is not only energetically unfavorable, but also generates metabolic intermediates that may contribute to downstream adverse myocardial remodeling.

### Cardiac aging

Cardiac aging is an intrinsic process that results in cellular and molecular changes that impair cardiac function. Due to the high energy demand of the heart, it is not surprising that age-related mitochondria defects are associated with diminished cardiac function^[55]^. Many factors contribute to the reduced energetic capacity of cardiac mitochondria during aging, including mutations and deletions in the mitochondria genome, increased ROS production, inflammation, altered mitophagy, and dysregulation in proteostasis and mitochondrial biogenesis^[56]^. It has been documented that the activity of mitochondrial respiratory chain complexes and proteins involved in mitochondrial metabolism, including those in FA metabolism, declines with age in the heart^[57]^. In contrast, extracellular structural proteins and glycolytic pathways increase substantially with aging^[58]^. Additionally, studies have consistently shown that age-related increases in mitochondrial ROS result in deleterious lipid and protein oxidation and accumulation of mtDNA that impairs the mitochondrial respiratory efficacy and further increases ROS production, forming a vicious cycle^[59–61]^. Aging also likely contributes to diminished replication fidelity and quantity of mtDNA, which promotes the accumulation of dysfunctional mitochondria, leading to adverse outcomes^[62,63]^.

In humans and several model systems, evidence suggests that mitochondrial structure is disrupted by the aging process. A disrupted morphology of the mitochondria and loss of cristae in the aged inner mitochondrial membrane has been shown with electron microscopy^[64–66]^. Studies have also indicated reduction and remodeling of cardiolipin in aging mitochondria^[67,68]^. As cardiolipin is responsible for maintaining optimal mitochondrial function and structure through its role in maintaining the proton gradient, cristae curvature, and preventing apoptosis, its loss is detrimental to the cardiac bioenergetic milieu^[69,70]^.

Insulin-like growth factor signaling, the mammalian target of rapamycin (mTOR), and regulation of histone acetylation by sirtuins are among the regulatory pathways that modulate cardiac health and aging. Modification of these pathways during the aging process can trigger mitochondrial dysfunction to accelerate cardiac impairment. In humans, an age-dependent decrease in serum insulin-like growth factor 1 was shown to be correlated with an enhanced risk of heart failure^[71]^. Autophagy, protein translation, lipid synthesis, and ribosome biogenesis are just a few of the crucial processes that mTOR controls. In various model organisms, the mTOR inhibitor rapamycin is known to increase lifespan^[72,73]^ while remodeling the aged heart proteome to a more youthful composition associated with improved mitochondrial function and decreased abundance of glycolytic pathway proteins. These findings might point to proteomic and metabolic remodeling as a mechanism behind the cardiac functional benefits granted by mTOR inhibitors^[58]^.

Increased mitochondrial protein hyperacetylation has been observed in myocardial tissues from the failing hearts of both humans and animals. It has been demonstrated that protein hyperacetylation reduces the activity of the TCA cycle enzymes succinate dehydrogenase, pyruvate dehydrogenase, as well as the malate-aspartate shuttle^[74]^. Acetylation-mediated impairment of the malate-aspartate shuttle limits the transfer of cytosolic NADH into the mitochondria and alters the cytosolic redox state^[75]^. Increased myocardial short-chain acyl-CoA content may contribute to increased protein acetylation; reduced protein deacetylation by sirtuins is another probable cause [Figure 1]^[76,77]^. Given their regulation by intermediate metabolites, sirtuins have been suggested to act as sensors of metabolic flux, and they are known to play a role in metabolic heart disease, natural aging, and heart failure^[78,79]^. Three of the seven mammalian sirtuins reside in the mitochondria (SIRT3, SIRT4, and SIRT5), with SIRT3 acting as the main driver of deacetylation^[80]^. Downregulation of SIRT3 has been observed in aged and failing hearts and may be attributable to decreased NAD^+^ levels or NAD^+^/NADH ratios, as low NAD^+^ levels inhibit SIRT3, leading to mitochondrial protein hyperacetylation and dysfunction^[75,81]^. Sirtuins also help mediate apoptosis signaling and reduce ROS by regulating antioxidant enzymes such as manganese superoxide dismutase (MnSOD) and catalase^[82,83]^. Genetic models have shown that SIRT3 knockout results in a reduction in complex I and III of the electron transport chain and decreased FA oxidation leading to a more glycolytic state^[83]^.

### Mitochondrial quality control: biogenesis, dynamics, and mitophagy

**Figure 2.**
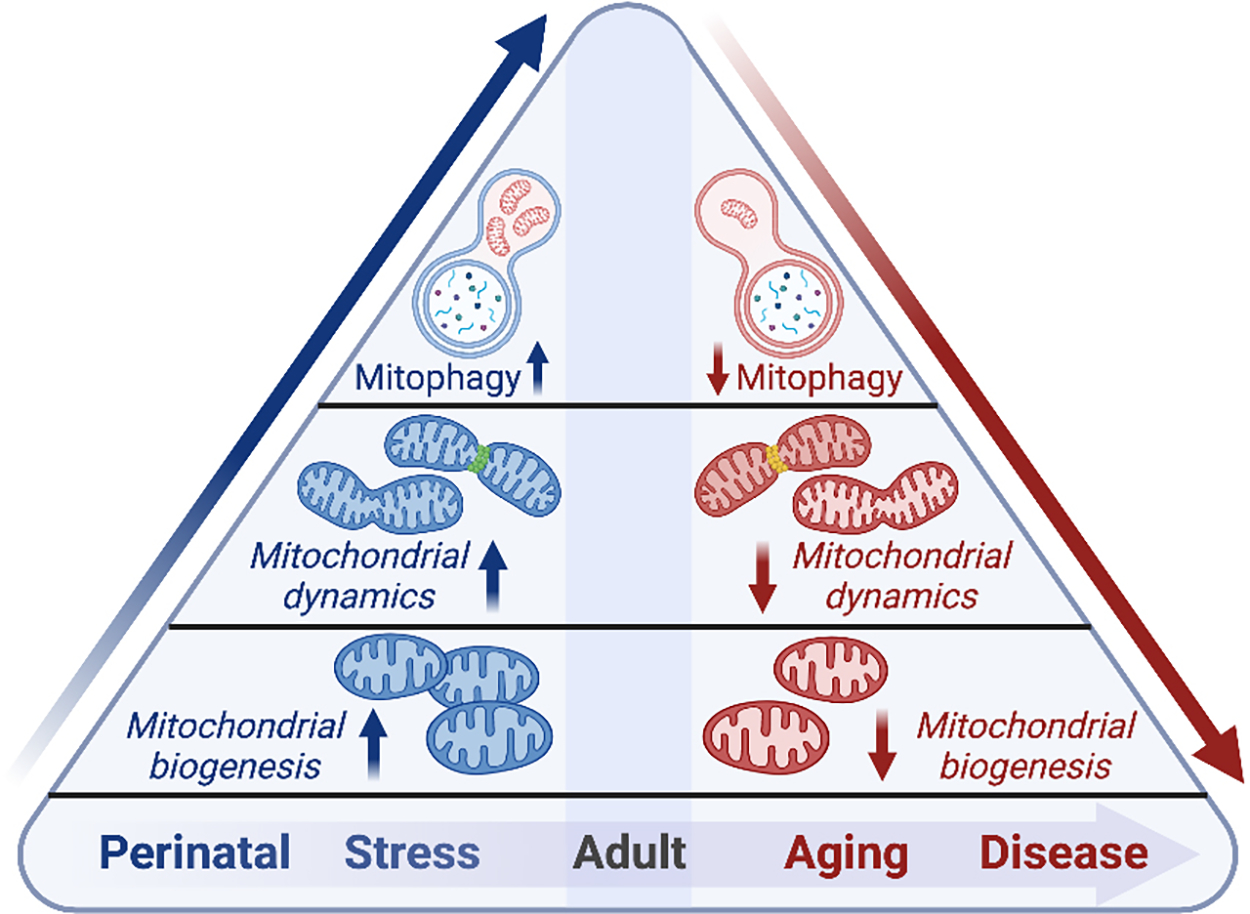
Mitochondrial quality control mechanisms are activated during development and in periods of stress, while aging and disease are associated with diminished mitochondrial quality control. See Section “Mitochondrial quality control: biogenesis, dynamics, and mitophagy” for further details.

Mitochondria are dynamic organelles that respond to physiological and pathological cues to confer adaptation to intracellular stresses and cellular energetic demand. As cardiac cells are post-mitotic and have limited capacity to proliferate during adulthood, maintenance of cardiomyocyte mitochondria is essential to prevent energetic failure and the accumulation of ROS. Mitochondrial fitness is maintained through QC mechanisms involving mitochondrial dynamics such as fission and fusion, biogenesis, and mitochondrial clearance or mitophagy [Figure 2]. The diversity of mitochondrial QC processes varies based on the energetic demand of the cell and cell-type specific regenerative capacity^[84]^. Here, we delineate the importance of specific mitochondrial QC mechanisms such as fission/fusion, biogenesis, and mitophagy, and discuss the contribution of impaired QC in the context of cardiac disease.

Mitochondrial biogenesis refers to the synthesis of a new organelle. The heart relies on mitochondrial biogenesis to adapt to an increase in energetic demand, such as during the transition from embryonic to post-natal growth^[85]^. Mitochondrial biogenesis is transcriptionally regulated by the PGC-1 family of transcriptional coactivators^[86]^. In the heart, gain and loss of function studies have revealed distinct and complementary roles for Pgc-1 coactivators in biogenesis and mitochondrial OXPHOS gene expression. Cardiomyocyte-specific Pgc-1 overexpression caused dilated cardiomyopathy (DCM) characterized by a massive increase in mitochondrial population^[87]^. Similarly, over-expression of Pgc-1α either in the post-natal period or in adulthood also caused DCM^[88]^. Interestingly, cardiac-specific deletion of Pgc-1α also caused a DCM phenotype in mice^[89]^. In contrast to Pgc-1α, Pgc-1β whole-body knockouts had no obvious cardiac phenotype under unstressed conditions^[90]^ but did develop systemic insulin resistance due to abnormal liver function^[91,92]^. In addition to Pgc-1α and β, Pgc-1-related coactivator is another regulator of mitochondrial biogenesis that has been shown to be essential for embryonic development^[93]^, but its role in the mature heart is less clear.

Impairments in mitochondrial morphology, content, and function are hallmarks of the diseased heart. Most of the data related to the changes in mitochondrial biogenesis in human heart failure have relied on the quantification of PGC-1α transcripts. Gupte *et al*. reported a 1.3-fold decrease in PGC-1α in human end-stage heart failure samples^[94]^. In mice, PO-induced heart failure is associated with reduced Pgc-1α expression^[95,96]^. Similarly, deletion of Pgc-1β precipitated cardiac dysfunction following PO in mice^[97]^. Contrary to these studies, others have reported increased or unchanged protein expression of PGC-1α in human failing hearts or in PO-induced heart failure in mice^[98–100]^. These discrepancies could be related to differences in the duration and the severity of heart failure as well as the type of medication the patients were taking. In addition to PGC-1 coactivators, mitochondrial biogenesis involves other transcriptional regulators including nuclear respiratory factor 1/2 (NRF1/2) and mitochondrial transcription factor A (TFAM)^[101]^. Cardiac ablation of Tfam resulted in mice either dying in the first week of life or three months after weaning with a DCM phenotype^[102]^. These findings underscore the importance of mitochondrial biogenesis during the perinatal to post-natal period, a period of high energy demand and a substrate switch from glucose to FAO.

Mitochondrial biogenesis is also activated in the setting of diabetic cardiomyopathy. Our group reported elevated Pgc-1α mRNA expression levels, increased mitochondrial number, and elevated mtDNA in the heart of leptin receptor deficient (db/db) mice^[6]^. Similarly, mtDNA and Pgc-1α, but not Pgc-1β, were elevated in the hearts of insulin resistant UCP-diphtheria toxin A mice^[103]^. Interestingly, the increase in cardiac Pgc-1α expression in these mice was completely abolished by genetic deletion of Pparα. These results suggest that there is a coordinated increase in mitochondrial biogenesis and FAO gene transcription in the insulin resistant diabetic heart, which is primarily regulated by Pparα. Aside from genetic models of obesity and diabetes, high fat feeding for ten weeks similarly enhanced cardiac Pgc-1α mRNA expression and increased mitochondrial content in mice^[103]^. Contrary to obesity and type II diabetic mouse models, Pgc-1α mRNA does not change in the hearts of Akita mice^[104]^, which are a monogenic model of type 1 diabetes wherein mutation in the insulin 2 gene leads to improper folding of the insulin protein, resulting in pancreatic toxicity, reduced β-cell mass, and reduced insulin secretion. In contrast, a reduction in mitochondrial biogenesis was reported in the heart of mice treated with streptozotocin to induce type I diabetes^[104–106]^.

Mitochondrial dynamics refers to the morphological change in mitochondrial shape during the cycle between fusion and fission states^[107–110]^. In mammals, mitochondrial fusion involves mainly three proteins: MFN1, MFN2, and OPA1^[111,112]^. Mitochondrial fission, on the other hand, involves two main proteins in mammals: Dynamin-related protein 1 (DRP1) and Mitochondrial fission protein 1 (FIS1)^[113–115]^. In addition to these core proteins, mitochondrial fission and fusion are facilitated by additional proteins and involve both transcriptional and post-transcriptional mechanisms reviewed elsewhere^[63]^.

The ultrastructure of the heart consists of compacted myofilaments with mitochondria located in between and closer to the myosin heads and endoplasmic reticulum to couple energy production to utilization and contraction. This well-organized compartmentalization makes it difficult for mitochondria to regularly change shape, especially in the adult heart^[63]^. However, mitochondrial dynamics play a central role in the perinatal period of heart growth. The disruption of fusion through the expression of a mutant Mfn2 in the heart perinatally but not postnatally was shown to result in cardiomyopathy^[116]^. In contrast, cardiomyocyte specific Mfn1 deletion caused mitochondrial fragmentation and conferred protection against ROS, with no apparent deleterious functional consequences^[117]^. The lack of cardiac dysfunction in these mice may be due to compensation by Mfn2. Indeed, tamoxifen-inducible cardiomyocyte Mfn1/Mfn2 double knockout mice develop progressive DCM^[118,119]^. Moreover, deletion of the fission protein Drp1 in cardiomyocytes germline or postnatally caused embryonic lethality or death shortly after birth, whereas tamoxifen-inducible deletion caused DCM^[120–122]^. Contrary to Mfn1/Mfn2 or Drp1 cardiac knockouts, tamoxifen-inducible ablation of both fission and fusion (Mfn1/Mfn2/Drp1 triple deletion) in the adult heart produced a less deleterious cardiac phenotype characterized by concentric cardiac hypertrophy; however, the animals still died about six months post-tamoxifen^[123]^. Aside from Mfn1/2 and Drp1, altered Opa1 processing in cardiomyocytes has been associated with mitochondrial fragmentation leading to the development of heart failure and early death in mice^[124]^. These studies highlight the importance of maintaining a balance between fusion and fission and provide evidence that proper mitochondrial dynamics are crucial for preserving function in both the developing and the adult heart.

An imbalance in mitochondrial dynamics is associated with several cardiovascular diseases^[125]^ including ischemia-reperfusion^[126–129]^, diabetic and non-diabetic cardiomyopathies^[130–135]^, cardiac hypertrophy and heart failure (HF)^[136–141]^. The mechanisms underlying mitochondrial remodeling during cardiac disease are not yet fully characterized, but a recent study by Tsushima *et al*. implicated lipid overload in the induction of mitochondrial fission in the heart^[134]^. Whether impaired mitochondrial dynamics is the cause or the consequence of cardiac pathology remains to be determined, but numerous genetic studies in mice have shown that altering mitochondrial dynamics can cause cardiomyopathy and heart failure. A subsequent section of this review is dedicated to the contribution of mitochondrial dynamics to cardiac structural remodeling.

Mitophagy refers to the selective clearance of mitochondria. As is the case for mitochondrial biogenesis and dynamics, mitophagy is highly regulated. It is also often coupled to mitochondrial biogenesis and occurs following mitochondrial fission. Mitophagy requires intact macro-autophagy/lysosomal function but may also occur independently of autophagy in some cases. Mitophagy proteins Parkin and PINK1 are essential players in the process of mitochondrial clearance. PINK1 is translocated to depolarized or stressed mitochondria and recruits Parkin to ubiquitinate proteins in the outer membrane, providing the signal for the recruitment of autophagosomes to eliminate the organelle^[142]^. This ubiquitin-dependent mitophagy has been extensively studied *in vitro* in the presence of drugs that reduce mitochondrial membrane potential. Another form of mitophagy mediated by mitophagy receptors but independent of ubiquitination of mitochondrial proteins has also been described^[143]^. There are currently four main mitophagy receptors described: SQSTM1 (also called p62), optineurin (OPTN), BNIP3, and FUNDC1, all of which contain the LC3 recognition domain and allow the tethering of mitochondria to autophagosomes^[144–146]^.

Genetic strategies have informed us about the importance and the redundancy of mitophagy proteins in the heart. For example, germline deletion of Pink1 led to the development of cardiac hypertrophy and dysfunction with age^[147]^. Parkin, on the other hand, has been shown to minimally affect the heart at baseline^[148,149]^. In contrast, absence of Parkin perinatally or during ischemic stress had deleterious consequences^[116,148,150]^. Contrary to Pink1 and Parkin, mitophagy receptors have been less studied. Although their association with mitochondria has been previously established, especially under conditions of stress, their direct role in mitophagy in the heart has not been extensively studied apart from FUNDC1. Cardiac-specific Fundc1 deletion caused mitochondrial elongation and led to the development of heart failure, which suggests that this receptor may facilitate both fission and mitochondrial clearance^[151]^. Together, these studies imply that mitophagy is dispensable in the adult heart under basal conditions, but may be necessary in response to stress.

Indirect evidence for the involvement of mitophagy in cardiac disease has been provided by the Otsu group, who showed that alteration of lysosomal degradation of mtDNA by deleting cardiomyocytes lysosomal deoxyribonuclease II caused cardiomyopathy and death after PO^[152]^. Failure to eliminate mtDNA led to the induction of cardiac inflammation. A similar reduction in mitophagy and accumulation of mtDNA was observed in the hearts of a mouse model of type 2 diabetes post-infarction^[153]^, and it has been shown that total Bnip3 expression was elevated in the hearts of heart failure patients^[154]^. Diet-induced obesity has been shown to result in a sustained elevation of cardiac mitophagy, during which autophagy was initially elevated but then declined at later stages^[155]^. This autophagy-independent form of mitophagy involved Ulk1 and Rab9 and was shown to play a protective role against diet-induced cardiomyopathy. Taken together, these studies provide compelling associations between impaired mitophagy and cardiac disease.

## MITOCHONDRIAL DYSFUNCTION DRIVES CARDIAC REMODELING

### Accumulated metabolic byproducts

The heart consumes more ATP than any other organ in the body, and without a substantial local energy storage system, it relies on substrate uptake from the circulation to produce ATP primarily through OXPHOS. Thus, a mismatch between substrate uptake and oxidation can have devastating consequences on heart structure and function. This is evident when substrate uptake is compromised, such as for glucose in the setting of insulin resistance, wherein the heart is forced to almost exclusively utilize fatty acids^[156]^. Similarly, when FA oxidation is compromised, the heart switches to glucose oxidation or the oxidation of alternative substrates, such as in the failing heart^[157,158]^. It is accepted that a defect in mitochondrial function may contribute to altered substrate oxidation in the diseased heart^[159–163]^; thus, progressive failure of mitochondrial respiratory machinery may result in the accumulation of backlogged metabolites and metabolic intermediates. Many of these compounds are unstable and prone to oxidation or other modifications that form toxic byproducts, negatively feeding back on mitochondrial function and further driving cardiac structural changes in a cyclic cascade degenerative remodeling.

**Figure 3.**
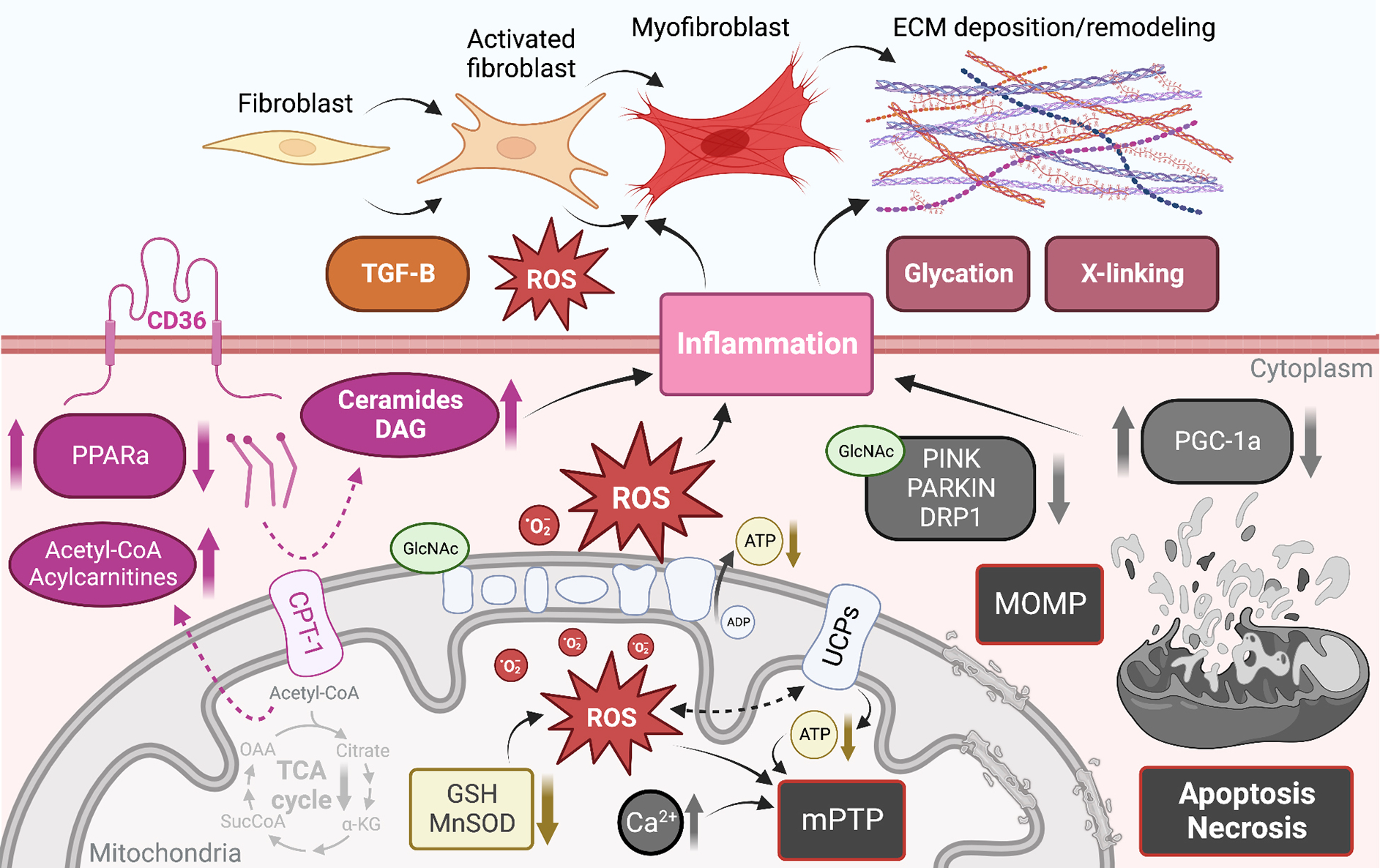
Mitochondrial dysfunction and downstream consequences trigger and exacerbate myocardial structural remodeling. ECM: Extracellular matrix; ROS: reactive oxygen species; TGF: transforming growth factor beta; PPAR: peroxisome proliferator-activated receptor; CPT1b: carnitine palmitoyltransferase 1b; TCA: tricarboxylic acid; DAG: diacylglycerol; GSH: glutathione; mPTP: mitochondrial permeability transition pore; UCP: uncoupling protein; MOMP: mitochondrial outer membrane permeabilization; PGC-1: peroxisome proliferator-activated receptor γ co-activators 1; GlcNAc: O-GlcNAcylation. See Section “MITOCHONDRIAL DYSFUNCTION DRIVES CARDIAC REMODELING” for further details.

A defect in cardiac mitochondrial FA oxidation coupled with an increase in FA uptake causes the accumulation of toxic lipid species [Figure 3]^[164]^. To directly test the implication of cardiac lipid overload on contractile function, several mouse models of cardiac lipotoxicity have been generated^[164]^. Cardiac hypertrophy, diastolic dysfunction, apoptotic cell death, cardiac fibrosis, and development of heart failure have all been observed after genetic manipulation of FA uptake or oxidation in the heart^[165–171]^. In addition, mice with leptin or leptin receptor deficiency have been used as models of lipotoxicity, with early studies establishing a link between cardiac TG accumulation and diastolic dysfunction in these mice^[172,173]^. Whether the early diastolic dysfunction associated with cardiac lipid accumulation is a consequence of cardiac fibrosis has not been directly tested. However, studies have shown that exposing cardiomyocytes to the saturated lipid palmitate *in vitro* induced cell death^[174,175]^ and led to replacement fibrosis *in vivo*^[166,167,174]^. Similarly, defects in mitochondrial FA oxidation and increased FA uptake can lead to the formation of signaling lipid species that are known to cause cell death. This is the case for the sphingolipid intermediates ceramides, which are elevated in mouse models with enhanced cardiac FA uptake [Figure 3]^[167,174]^. Ceramides have been shown to induce cell death in cardiomyocytes *in vitro*^[176]^, but whether they cause cell death and replacement fibrosis in the heart *in vivo* has yet to be demonstrated.

As mentioned above, a common feature of altered FA oxidation in the heart is the accumulation of lipids and the development of cardiac hypertrophy. This has been recapitulated genetically by directly inhibiting the mitochondrial transport of FA through the deletion of Cpt1b in the heart [Figure 3]. Heterozygous Cpt1b knockout mice develop cardiac hypertrophy, increased fibrosis, and die within two weeks following transverse aortic constriction^[177]^. Similarly, conditional deletion of Cpt1b in skeletal and cardiac muscle caused massive cardiac hypertrophy and reduced survival due to the development of congestive heart failure, but cardiac fibrosis was not assessed in these mice^[178]^. Similar findings were observed in mice with defective beta-oxidation or lacking the master regulator of FA metabolism Ppparα^[179–181]^, where the accumulation of cardiac fibrosis in Pparα null mice developed at a later stage than the other alterations observed in these animals^[180]^. Together, these studies associate cardiac lipid accumulation with cardiac fibrosis, but a causal relationship has not been demonstrated; in fact, another study detected cardiac lipid accumulation in the absence of cardiac fibrosis in the hearts of type 1 Akita mice^[182]^.

There are multiple mechanisms underlying mitochondrial dysfunction-induced cardiac fibrosis (discussed subsequently). However, few studies have explored the pro-fibrotic signaling by cardiac lipids. As mentioned above, lipid overload in cardiac cells induced ER stress and caused cardiomyocyte apoptosis. Loss of cardiomyocytes has been consistently observed in mice with cardiac lipid overload and coincides with the appearance of replacement fibrosis^[166,167,174,176]^. The mechanisms implicated in lipid-induced cardiomyocyte apoptosis may involve an increase in lipid peroxide, enhanced iNOS expression and NF-κB activation^[183]^. Excess polyunsaturated fatty acids such as linoleic, linolenic, or arachidonic acids have been shown to increase collagen I/III ratio in the mouse myocardium, leading to stiffening characterized by impaired transmitral flow, which is a hallmark of early diastolic dysfunction^[184]^. Diacylglycerol, which is known to activate Pkc, is elevated in the hearts of streptozotocin-induced diabetic mice^[185]^, and Pkc was shown to activate Tgfβ and extracellular matrix (ECM) synthesis and fibrosis in multiple cell types [Figure 3]^[186]^. Aside from cardiomyocytes, excess lipids induce ER stress, inflammasome activation, and apoptosis in cardiac fibroblasts^[184,187]^. Finally, interventions that reduced cardiac lipid accumulation or lipoapoptosis were efficacious in reducing cardiac fibrosis in obese diabetic rodents^[183,188,189]^.

Nutrient excess and the accumulation of metabolic byproducts have been increasingly recognized to contribute to post-translational protein modification (PTPM). Until the mid-1980s, PTPM was only known to occur extracellularly^[190]^, but it is now widely acknowledged that nuclear, cytosolic, and mitochondrial proteins are also susceptible, and that these modifications are important regulators of protein function that may result in significant alterations in mitochondrial and whole heart function^[191–195]^. The most widely recognized form of intracellular PTPM is known as O-GlcNAcylation and occurs when β-N-acetylglucosamine is appended to the serine and threonine residues of proteins *via* an O-linkage. O-GlcNAcylation signaling is regulated in part *via* the hexosamine biosynthetic pathway, and thus combines inputs from glucose, amino acid, fatty acid, and nucleotide metabolism^[196]^. O-GlcNAcylation is now recognized to affect signal transduction, calcium handling, apoptosis, autophagy, proteasomal degradation, transcription factors, and chromatin modifiers. Additionally, O-GlcNAcylated proteins have been identified in complexes I, II, and III of the mitochondrial respiratory chain. In particular, increased O-GlcNAcylation of mitochondrial complex I subunit A9 decreased the respiratory activity of the complex, which was rescued upon removal^[192]^. Alterations to mitochondrial fission have also been reported with O-GlcNAcylation of DRP1 [Figure 3]^[197]^.

In contrast to O-GlcNAcylation, which occurs intracellularly, advanced glycation end products (AGEs) are a family of compounds formed extracellularly by non-enzymatic glycation of proteins and lipids by reducing sugars such as glucose and ribose. Interest in AGEs has grown rapidly in recent years, as they exhibit a wide range of chemical, cellular, and tissue-level effects that have been implicated in numerous diabetes-related complications in both humans and animal models across a range of cell and tissue types^[198–200]^. While tissue and plasma AGEs are well-known to accumulate progressively with aging, the rate and extent of AGE accumulation in the heart are dramatically accelerated during diabetic cardiomyopathy and chronic heart failure^[201]^ due to excess circulating glucose and glycolytic byproducts. AGEs have widely been shown to drive pathological structural remodeling of the myocardial interstitium *via* irreversible cross-linking of ECM collagens and proteoglycans [Figure 3], which leads to myocardial stiffening and functional decline^[202]^. *In vitro* models have also shown that cardiac cells treated with AGEs exhibit significantly decreased respiratory capacity^[203]^, as well as dose- and time-dependent increases in ROS generation and apoptosis^[204,205]^.

Beyond their direct toxicity and adverse modification of ECM structure, AGEs also initiate a cascade of signal transduction *via* association with the receptor RAGE that further drives oxidative stress, inflammation, cell dysfunction, and apoptosis^[206]^. A study of RAGE knockout mice noted mRNA transcripts and protein expression levels associated with increased antioxidant defenses, improved mitochondrial dynamics, and improved autophagy-lysosomal flux (*via* SOD, Drp1 and Fis1, and cathepsin-L activity, respectively), which were found to partially protect against diet-induced pathological changes^[207]^. These results and many others suggest that the AGE/RAGE axis may play a key role in the development of cardiac mitochondrial dysfunction and drive a cyclic cascade of cardiac structural remodeling in the context of metabolic disease, diabetic cardiomyopathy, and chronic heart failure^[208]^.

### Oxidative stress

Numerous studies have identified molecular mechanisms linking increased mitochondrial ROS production and the associated oxidative stress with cellular- and tissue-level remodeling of the heart. Myocardial ECM remodeling is chiefly characterized by the diffuse deposition of excessive extracellular collagen, which is typically quantified by an increase in the percentage of total myocardial tissue occupied by collagen fibers^[209]^. Disproportionate accumulation of collagen between cardiomyocytes (interstitial or reactive fibrosis) and replacement of apoptotic or necrotic cardiomyocytes with extracellular collagen (replacement or reparative fibrosis) both contribute to myocardial fibrosis and are consistently associated with cardiac diastolic dysfunction^[210,211]^. Additionally, post-translational modification of extracellular collagen is increasingly recognized to contribute to pathophysiological ECM remodeling and impaired cardiac function^[212,213]^.

Activated fibroblasts and myofibroblasts are recognized as the primary cellular drivers of myocardial fibrosis^[214]^; however, under conditions of stress, cardiomyocytes and immune cells, which produce substantial ROS, may also acquire a fibrogenic phenotype^[214]^. Mitochondrial ROS production is increased in activated fibroblasts and differentiated myofibroblasts, which exhibit enhanced expression of the ROS-producing NADPH-oxidase Nox4^[215]^. The additional ROS produced by these fibrogenic cells likely further regulates their pro-fibrotic action both by activating ECM gene transcription and by regulating post-translational modifications of ECM collagens [Figure 3]^[216,217]^. One redox-relevant PTPM of the ECM is the hydroxylation of collagen proline and lysine residues, which induces a conformational change within the collagen helix structure that leads to the formation of supramolecular fibrillar structures while conferring resistance to proteolytic degradation, both of which impair normal collagen turnover^[218–220]^. Additionally, ROS are believed to be involved in the formation of covalent disulfide bridges within numerous ECM protein domains; however, relatively little is known about the enzymes regulating the formation and cleavage of redox-sensitive ECM disulfide bridges^[221,222]^.

TGFβ1 is a fibrogenic growth factor that directs differentiation of fibroblasts into myofibroblasts and controls ECM production; it has been shown that ROS mediate TGFβ1-induced activation of fibroblasts and differentiation into myofibroblasts [Figure 3]^[223,224]^. TGFβ1 has also been shown to stimulate Nox4 expression, which produces additional ROS, forming a detrimental feedback cycle^[225,226]^. In addition to their effects on collagen synthesis and modification, ROS also substantially regulate the synthesis and activity of matrix metalloproteinases involved in ECM degradation and remodeling, which are generally secreted in an inactive form and has been shown to be activated post-translationally by ROS^[227,228]^. Thus, ROS are crucial regulators of ECM quantity and quality, as they exert both matrix deposition, modification, and degradation effects.

In addition to their fibrogenic actions, ROS have been shown to activate numerous canonical cell-signaling kinases and transcription factors involved in cardiac structural remodeling through modulation of protein and ion homeostasis, apoptosis, and growth pathways^[229,230]^. These effects have been shown to depend on several redox-sensitive kinases, such as PKC, MAPK, NF-κB, and PI3K-PKB/Akt^[231–233]^. In fact, it has been shown that PI3K is required for H_2_O_2_-induced cardiac hypertrophy^[234]^. Furthermore, activation of hypertrophic versus apoptotic kinase signaling pathways have been shown to be H_2_O_2_-concentration dependent: low H_2_O_2_ concentrations (10–30 uM) increased Erk1/2 (but not Jnk, p38 kinase, or Pkb) activity and increased protein synthesis without affecting survival, while higher concentrations (100–200 uM) activated Jnk, p38 kinase, Pkb, and increased apoptosis, with both apoptosis and necrosis observed at still higher concentrations (300–1000 uM)^[235]^. ROS also play an important role in G protein-coupled hypertrophic stimulation by angiotensin and adrenergic stimulators^[236,237]^, the latter involving oxidative modulation of Ras^[238]^.

### Mitochondrial quality control

As discussed above, maintenance of a dynamic mitochondrial population, including fission and clearance, is considered a protective mechanism during myocardial stress. Alteration in mitochondrial fission and clearance leads to the accumulation of damaged organelles that produce excessive ROS, exacerbating cardiac injury. In the adult heart, mitochondrial dynamics and mitophagy are minimal and deletion of proteins involved in mitochondrial dynamics or mitophagy has minimal effect under basal conditions. However, the necessity of these processes becomes evident during aging or in response to stress. While the causal relationship between mitochondrial QC and cardiac remodeling has not yet been directly investigated, indirect evidence suggests that the accumulation of dysfunctional mitochondria may exacerbate cardiac fibrosis through mechanisms involving ROS and inflammasome activation. Consistent with this idea, Pink null mice exhibit enhanced oxidative stress and fibrosis that was exacerbated with aging^[147]^. In contrast, Pink1 overexpression in a rat model of HFpEF stimulated mitochondrial fission and prevented cardiac fibrosis^[139]^. Similarly, deletion of Parkin in the adult mouse heart had no effect but mitigated excessive mitophagy and prevented cardiac remodeling and replacement fibrosis in the context of Drp1 deletion^[149]^. Similarly, impaired mitophagy *via* Ulk1 deletion exacerbated fibrotic remodeling after transverse aortic constriction and high-fat feeding in mice^[155,239]^. Perturbation of mitochondrial dynamics also resulted in the accumulation of cardiac fibrosis. Lack of the mitochondrial fission factor Mff led to the development of cardiomyopathy associated with fibroblast proliferation and replacement fibrosis^[240]^. Although the authors found increased apoptotic cell death in Mff knockout mice, they proposed that cell death alone did not account for the extent of fibrosis, suggesting additional mechanisms. Since Mff is a receptor for Drp1, knockout of Drp1 in the adult heart produced a similar cardiomyopathy phenotype characterized by the accumulation of substantial fibrosis^[122]^. Surprisingly, fibrosis is absent in Mfn1/Mfn2 double mutant mice, suggesting that altered mitophagy, rather than dynamics, may be causal for the development of fibrosis. The mechanisms by which altered mitophagy leads to cardiac fibrosis are not well understood but likely involve apoptotic cell death, oxidative damage, and immune cell infiltration. Indeed, impairing lysosomal degradation of mtDNA led to the activation of toll-like receptor 9 (Tlr9), which in turn activated immune cell infiltration in the heart^[152]^. Treatment of cardiac-specific DNase 2a knockout mice with a Tlr9 inhibitor or deletion of Tlr9 attenuated cardiac fibrosis^[152]^. These results suggest that either excessive or stalled mitophagy leads to cardiomyocyte death, oxidative stress, mtDNA accumulation, and inflammation, all of which contribute to significant fibrotic remodeling of the heart [Figure 3].

### Mitochondrial-mediated apoptosis and necrosis

Mitochondria are centrally involved in cell death pathways, and as such, mitochondrial health has a direct bearing on myocardial structure and function. The mitochondrial cell death pathway, which includes both apoptosis and necrosis, is activated by numerous factors, including nutrient deprivation, disrupted oxygen availability, excessive oxidative stress, nitrosative stress, proteotoxic stress, DNA damage, or elevated cytoplasmic or mitochondrial Ca^2+[241,242]^. Cardiac contractile tissues must sustain persistently high energetic demand to maintain numerous essential cellular processes such as ion transport, sarcomere function, and Ca^2+^ homeostasis. Thus, diminished mitochondrial respiratory capacity associated with the progression to heart failure is thus typically followed by secondary dysregulation of cardiac Ca^2+^ and ion handling and reduced contractile function, resulting in a cyclic cascade that activates cell death pathways.

Mitochondrial-mediated necrosis is associated with Ca^2+^-triggered opening of the mPTP in the inner mitochondrial membrane^[243]^, which may be dependent on cyclophilin D^[244]^. High Ca^2+^ is believed to be the proximal initiator of mPTP opening, and this process is known to be potentiated by oxidative stress and depletion of ATP and ADP [Figure 3]^[245]^. While it has since been disproven that subunits of ATP synthase are components of the mPTP^[246–248]^, it is agreed that opening of the mPTP immediately dissipates the proton gradient across the inner mitochondrial membrane, halting ATP production, and allowing the rapid osmotic influx of water into the solute-rich matrix, resulting in severe mitochondrial swelling.

In contrast, mitochondrial-mediated apoptosis is driven primarily by mitochondrial outer membrane permeabilization, which releases several apoptogenic factors promoting cytosolic procaspase activation and leading to apoptotic cell death [Figure 3]. The BCL-2 family of proteins is understood to be the primary regulators of mitochondrial OMP and either promote or inhibit cell death based on their specific BCL-2 homology domains^[249]^. The pro-survival BCL-2 subfamily contains homology domains BH1-4, whereas the pro-death BCL-2 proteins are believed to contain only BH1-3; a third group contains only the BH3 domain and promotes cell death. These three subfamilies engage in complex interactions^[242]^ to regulate mitochondrially mediated apoptosis *via* OMP.

Given the lack of proliferative capacity of cardiomyocytes in the adult heart, cardiomyocyte cell death *via* either the apoptotic or necrotic pathway results in a diminished functional population of contractile and conductive units. While the fibrotic scar tissue that typically replaces lost cardiomyocytes serves to maintain the structural integrity of the heart, preventing catastrophic mechanical failure^[250]^, replacement fibrosis is considered a functionally adverse structural remodeling response associated with diminished systolic and diastolic function, as well as conduction abnormalities^[214]^.

## CARDIAC REMODELING ALTERS MITOCHONDRIAL BIOENERGETICS

While the mechanisms by which disrupted mitochondrial function may potentiate cardiac structural changes have been widely studied, as described above, fewer studies have explored the effects of myocardial structural remodeling on mitochondrial milieu and bioenergetic demand. However, the bidirectionality of structural and energetic remodeling cascades may prove particularly important during the transition and decompensation stages of heart failure, when the pathological mechanical and biochemical environment overwhelms disrupted energy systems beyond their compensatory capacity. Thus, future studies of the mechanistic progression to heart failure should consider altered mitochondrial function as both a cause and consequence of structural cardiac remodeling, such as hypertrophy and fibrosis.

Compensatory hypertrophy is believed to develop initially as an adaptive response to help maintain cardiac output and mitigate tissue-level stresses through thickening and stiffening of the ventricular walls^[41]^. However, along with increased energy demand due to chronic overload and decreased energy production capacity, hypertrophy progresses maladaptively and mechanistically contributes to the bioenergetic deficit precipitating heart failure. Additionally, it has been shown that the growth pathways involved in the development of cardiac hypertrophy directly regulate mitochondrial morphology and bioenergetic function, including regulation of TCA cycle and FAO enzymes^[251]^. Mitochondrial morphology is highly dynamic, and this functional plasticity endows the organelles with substantial adaptive capabilities. In healthy cardiomyocytes, mitochondria are abundant and contain intact membranes and clear cristae structures. In hypertrophic hearts subject to chronic overload, mitochondrial density is decreased, and the organelles may become swollen, elongated, and deformed, exhibiting ruptured membranes and irregular cristae structures^[252,253]^. These deformities reduce the biogenetic capacity of the mitochondria in the hypertrophic heart, which further exacerbates the detrimental effects of cardiac structural remodeling and may contribute significantly to the progression into the decompensated stage of PO-induced heart failure.

**Figure 4.**
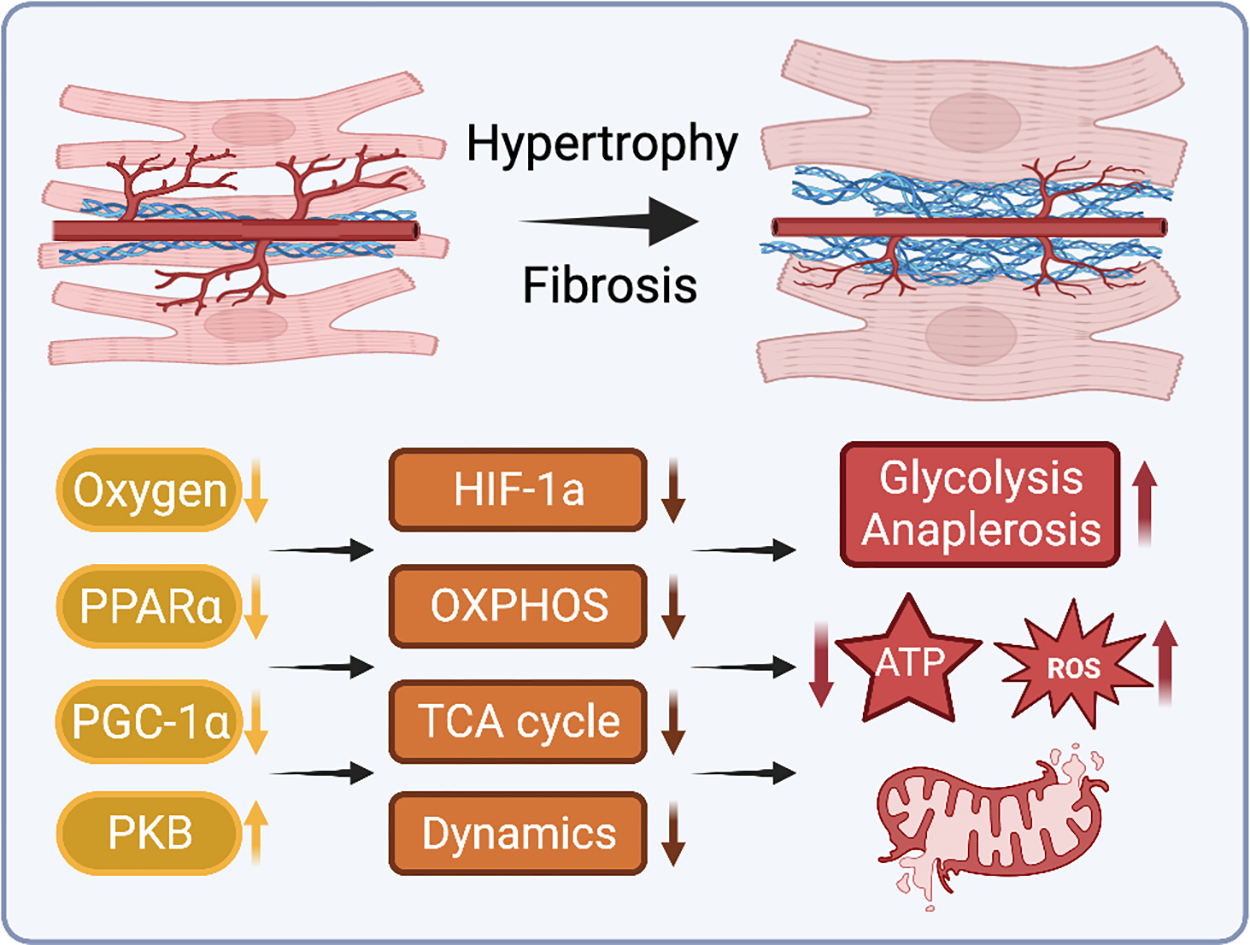
Myocardial structural remodeling impairs mitochondrial energetic supply and demand. ROS: Reactive oxygen species; HIF-1: hypoxia-inducible factor 1; OXPHOS: oxidative phosphorylation; TCA: tricarboxylic acid; PGC-1α: peroxisome proliferator-activated receptor γ co-activators 1α; PKB: protein kinase B. See Section “CARDIAC REMODELING ALTERS MITOCHONDRIAL BIOENERGETICS” for further details.

While studies have shown that growth pathways involved in cardiac hypertrophy significantly interact with metabolic regulatory processes, the molecular mechanisms by which hypertrophic remodeling alters mitochondrial function are not yet fully elucidated. PKB, also known as Akt, is a serine-threonine kinase that has been widely studied across diverse physiologic and pathologic settings^[254]^. Activation of Akt is strongly associated with hypertrophic cardiac growth; while adaptive in the short-term, persistent stimulation of Akt signaling is deleterious due to feedback inhibition of insulin receptor substrate, PI3K signaling, and GLUT4-mediated glucose uptake, and may help precipitate heart failure due to a mismatch between cardiac hypertrophy and angiogenesis [Figure 4]^[255–257]^. Furthermore, using mice with cardiomyocyte-specific constitutively activated Akt1 (caAkt) signaling, it has been shown that persistent activation of Akt directly alters cardiac mitochondrial bioenergetic function. Wende *et al.* observed selectively repressed expression levels of TCA cycle enzymes and proteins involved in OXPHOS, FAO, and mitochondrial biogenesis in caAkt hearts^[251]^. This was accompanied by a robust increase in left ventricular mass and contractile dysfunction at six weeks of age, as well as reduced functional measures of mitochondrial efficiency. Additionally, 18-week-old caAkt mouse hearts subject to ischemia-reperfusion showed decreased rates of glucose oxidation, palmitate oxidation, and myocardial oxygen consumption concomitant with increased glycolysis. A canonical pathways analysis also revealed that several mitochondrial metabolic and signaling pathways were differentially regulated in caAkt hearts, including Foxo1, Pparα, and Pgc-1α. Thus, hypertrophic factors such as Akt have a direct effect on mitochondrial bioenergetics and morphology.

Numerous animal studies of pressure-induced hypertrophy have reported reduced FAO rates during the compensated stages of hypertrophy preceding the onset of overt heart failure^[47,258,259]^. In addition to decreased fatty acid oxidative capacity, carnitine deficiency and reduced CPT-1 activity in cardiac hypertrophy may limit mitochondrial uptake and utilization of fatty acids^[260,261]^. Meanwhile, glucose oxidation has been variously observed to increase, remain unchanged, or decrease during cardiac hypertrophy and heart failure^[47,258,262,263]^. While decreased fatty acid and glucose oxidation in hypertrophy may be partially compensated by anaerobic glycolysis and increased anaplerotic flux through the TCA cycle, this is less energetically efficient and may contribute to energy insufficiency during the transition to heart failure [Figure 4]^[264]^.

Interstitial and perivascular fibrosis, as well as intramyocardial arterial thickening, often occur during the compensated stage of cardiac hypertrophy, increasing oxygen and substrate diffusion distances^[265,266]^. Without sufficient compensatory angiogenesis, limited oxygen availability in areas of substantial fibrosis may cause cardiomyocyte hypoxia and impair mitochondrial respiration, generating additional ROS, limiting aerobic substrate metabolism, and eventually resulting in tissue-level hypoxia and cardiomyocyte death [Figure 4]^[267]^. This response is in part mediated by HIF-1, which is involved in coordinating transcriptional programs upregulating enzymes involved in glycolysis and downregulating mitochondrial respiratory complex proteins^[268]^. This shifts cardiac bioenergetics away from efficient aerobic metabolism toward anaerobic pathways with substantially lower energetic yield^[269]^. Given the increased energetic demand of the hypertrophic heart, these conditions set the stage for a cyclic cascade of energetic mismatch.

In addition to global inhibition of aerobic metabolism *via* decreased oxygen and substrate transport, it has also been suggested that defects in the mitochondrial electron transport chain contribute to energy insufficiency during PO-induced hypertrophy. It has been observed that decreases in mitochondrial complex I State 3 respiratory capacity occurred concurrently with the development of systolic function, after the development of diastolic dysfunction, which began while mitochondrial function was still preserved^[270]^. This may suggest a mechanistic link between early cardiac structural remodeling associated with diastolic dysfunction and the subsequent development of mitochondrial dysfunction. Similarly, it has been observed that mitochondrial ROS production remained at normal levels during early compensated hypertrophy, only increasing with the onset of diastolic dysfunction and worsening with the progression to heart failure^[270]^, similarly suggesting that early structural remodeling may in fact drive subsequent alterations to mitochondrial function.

## PATHOGENIC CASCADES AND OPPORTUNITIES FOR THERAPEUTIC INTERVENTION

### Diabetic cardiomyopathy

Diabetic cardiomyopathy refers to the diabetes-specific cascade of structural myocardial remodeling and functional decline not attributable to other macrovascular conditions such as atherosclerosis or hypertension and is expected to affect more than 30 million people^[271]^. The abnormal cardiac structural and functional changes associated with diabetic cardiomyopathy are promoted by chronic hyperglycemia, hyperinsulinemia, and resistance to the metabolic actions of insulin in the heart^[272]^, with cardiovascular event risk quantifiably correlated to the level of glycemic control^[273]^. In a Swedish observational trial of 20,985 individuals with type 1 diabetes, each 1% rise in HbA1c was linked to a 30% increase in the risk of heart failure, independent of other risk factors^[274]^. Despite a drastic increase in the number of studies on diabetic cardiomyopathy in the last decade, optimal therapies to treat diabetic cardiomyopathy are still lacking. However, recent advances in the development of SGLT2 inhibitors have shown great promise in the treatment of diabetes-associated heart failure, as discussed further below.

In the early stages of diabetic cardiomyopathy, metabolic disturbances such as defective insulin signaling, excessive circulating insulin, impaired glucose uptake, elevations in myocardial fatty acid uptake, and mitochondrial dysfunction promote cardiac morphological and functional changes^[275]^. These combined metabolic abnormalities lead to pathophysiological changes including increased oxidative stress, reduced cardiomyocyte autophagy, inappropriate activation of the renin-angiotensin-aldosterone system, and maladaptive immune responses, which can all activate pro-fibrotic pathways resulting in cardiac stiffening and diastolic dysfunction^[276]^. Insulin resistance and dysregulated glucose homeostasis are not only detrimental to mitochondria *via* excessive ROS production and altered cardiomyocyte calcium handling but can also increase FFA flux through CD36, causing lipid accumulation. Excessive accumulation of intracellular FFA exceeding mitochondrial oxidative respiratory capacity results in lipotoxicity and eventually cardiomyocyte death and impaired cardiac function^[172,277,278]^. Alterations to cardiac structure are more pronounced in the later stages of diabetic cardiomyopathy and include cardiomyocyte apoptosis and necrosis, widespread interstitial and replacement fibrosis, increased ECM crosslinking, hypertrophy, and capillary microaneurysms^[279,280]^. Additionally, hyperglycemia increases the rate of advanced glycation end-product crosslink formation, further stiffening the myocardial matrix, increasing ROS production, and activating RAGE signaling pathways^[199]^. Specific mitochondrial-targeted therapeutics that may be used in the treatment of diabetic cardiomyopathy will be discussed subsequently, but improving systemic glycemic control remains a key factor in the prevention of diabetic cardiomyopathy and cardiovascular morbidity.

### Cardiac ageing

Approximately one in five individuals over 80 years old are at risk of cardiac dysfunction and heart failure, and among patients with congestive HF, arterial fibrillation, and coronary heart disease, over 70% are elderly^[281,282]^. Intrinsic cardiac aging is understood as the gradual progression of structural alterations and functional declines occurring with age independent of the prolonged exposure to canonical cardiovascular risk factors. The ‘free radical theory of aging’ suggests accumulated macromolecule oxidative damage as a hallmark of aging^[283]^. This paradigm is supported by observations that aged cardiomyocytes show increased markers of oxidative damage and associated abnormalities in mitochondrial structure, such as loss of cristae, enlarged organelles, and matrix derangement^[284]^. Additionally, age-dependent reductions in mitochondrial OXPHOS are correlated with a decline in the activity of ETC complexes I and IV, which may be driven by increased ROS generation and electron leakage. Furthermore, aged hearts exhibit four-fold increases in deletion frequency and point mutations in mtDNA, as well as increased mtDNA copy number^[285]^. Significantly downregulated autophagy is also widely observed in the aging heart; the inability to eliminate damaged cells and repair damaged organelles is particularly important in post-mitotic cells such as cardiomyocytes^[286]^. Therefore, therapeutic approaches that counteract age-related changes in mitochondrial QC, autophagy regulators, inflammation, and ROS generation would be potential targets to mediate the deleterious effects of cardiac aging.

### Heart failure (with preserved and reduced ejection fraction)

National Health and Nutrition Examination Survey data indicates that 6.5 million American adults were living with heart failure (HF) as of 2014, with this number expected to increase to over 8 million by 2030^[287]^. Among patients presenting with clinical heart failure, left ventricular ejection fraction (EF) has emerged as a clinically useful prognostic indicator^[288,289]^, where a bimodal distribution of EFs allows for patient classification into two HF phenotypes: heart failure with reduced ejection fraction (HFrEF; EF ≤ 40%) or heart failure with preserved ejection fraction (HFpEF). HFpEF has recently become the dominant form of heart failure worldwide alongside an aging population and concomitant with the increased prevalence of obesity, diabetes, and hypertension, which are all associated more strongly with HFpEF than with HFrEF^[290]^. Given the distinct etiology, pathophysiology, and specific therapeutic potential, it is essential that both heart failure phenotypes be well-characterized so that optimal diagnostic and treatment strategies may be developed and applied for each^[291–293]^.

While the diagnosis and treatment of HFpEF are complicated by heterogenous etiologies and pathogeneses, as well as limited tissue availability from HFpEF patients, recent studies have succeeded in identifying several distinguishing cellular- and tissue-level characteristics in myocardial structure and function consistent with clinical observation of HFpEF patients (i.e., concentric ventricular hypertrophy, diastolic dysfunction, and exercise intolerance)^[294]^. These observations include structural changes such as cardiomyocyte hypertrophy and interstitial fibrosis^[295–297]^, functional measures such as impaired myofibrillar relaxation and increased cardiomyocyte stiffness^[210,295–297]^, as well as increased oxidative stress and activation of pro-inflammatory and pro-fibrotic signaling pathways^[297,298]^. Additionally, it has been shown that cardiomyocyte mitochondria from HFpEF hearts exhibit structural and functional deficits involving redox imbalance, impaired mitochondrial dynamics, and QC, and reduced bioenergetic function^[299]^. Together, these changes result in deleterious activation of downstream signaling pathways that exacerbate pathological cardiac remodeling and inflammation, and drive a detrimental mismatch between cardiac mitochondrial metabolism and ATP production^[154,300–303]^.

It is well established that HFrEF is differentially associated with a loss of cardiomyocytes and myocardial contractile function compared to HFpEF. While it is understood that these processes are at least in part mitochondrially mediated, as discussed above, relatively few studies have directly compared alterations to mitochondrial function between HFpEF and HFrEF hearts. In the context of HFrEF, it has been shown that mitochondrial content, electron transport chain complexes, and oxidative capacity are maintained or even enhanced during compensated hypertrophy alongside normal or increased EF, but that these parameters decline in concert with reducing EF during the progression to HF^[47,154,304]^. A 2019 study from Chaanine *et al*. directly comparing mitochondrial characteristics between human HFpEF and HFrEF hearts showed that human HFrEF hearts displayed uniquely increased DRP1 expression and decreased expression of PGC-1α and COX IV, as well as increased mitochondrial fragmentation and cristae disruption compared to HFpEF hearts, and that these alterations were associated with activation of the FOXO3a-BNIP3 pathway^[154]^.

### Mitochondria as therapeutic targets in heart disease

Myriad therapeutics have been developed to aid in the prevention and treatment of heart disease over the last century. In this section, we will primarily focus on therapeutic targets involving mitochondria in the context of metabolic heart disease, cardiac aging, and heart failure. Human and animal data both suggest that reduced mitochondrial biogenesis and increased oxidative stress are hallmarks of several heart disease and heart failure phenotypes, including diabetic cardiomyopathy, cardiac aging, and heart failure. As such, therapeutics targeted at increasing mitochondrial biogenesis and reducing ROS are likely to prove beneficial in the treatment of various HF phenotypes^[305]^.

**Figure 5.**
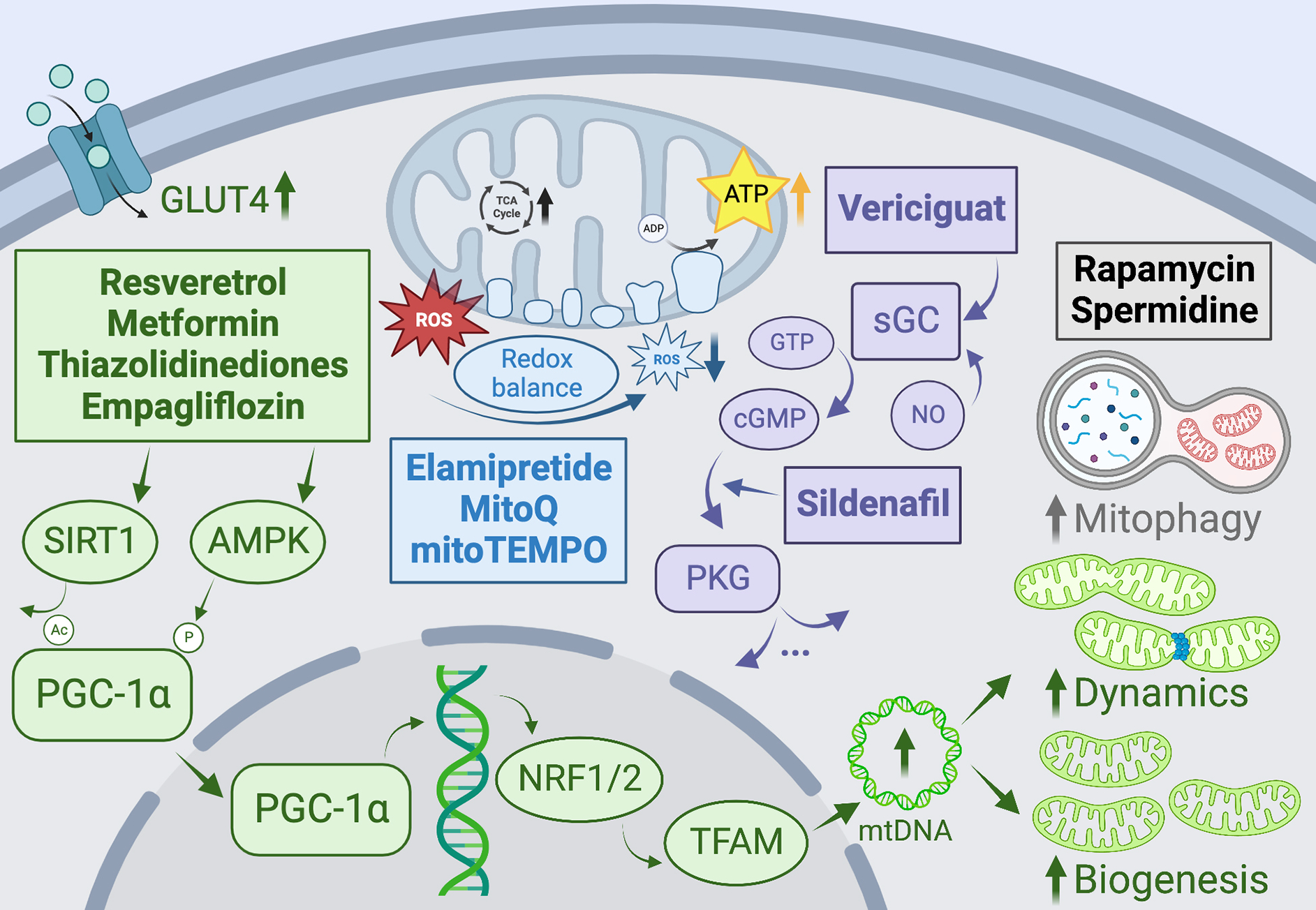
Promising pharmacologic therapies targeting mitochondrial pathways to ameliorate metabolic heart disease, aging, and heart failure. GLUT4: Glucose transporter type 4; PGC-1α: peroxisome proliferator-activated receptor γ co-activators 1α; TCA: tricarboxylic acid; ROS: reactive oxygen species; NRF1/2: nuclear respiratory factor 1/2; TFAM: mitochondrial transcription factor A; mtDNA: mitochondrial DNA; PKG: protein kinase G; cGMP: cyclic guanosine monophosphate; sGC: soluble guanylyl cyclase; GTP: Guanosine triphosphate. See Section “Mitochondria as therapeutic targets in heart disease” for further details.

One promising pharmacological avenue to stimulate mitochondrial biogenesis is through AMP-activated protein kinase (AMPK)^[299,306]^. AMPK is a highly conserved regulator of energy homeostasis and metabolism that is known to activate PGC-1α, the transcriptional coactivator considered to be the master regulator of mitochondrial biogenesis^[277]^. AMPK has been shown to increase mitochondrial biogenesis *via* both direct phosphorylation of PGC-1α and activation of NRF1/TFAM [Figure 5]^[307–309]^. Several widely used cardioprotective therapies have been suggested to target AMPK activation indirectly, such as metformin, telmisartan, and thiazolidinediones^[305,310,311]^. For example, metformin and thiazolidinediones improve systemic and tissue insulin sensitivity, improving cardiomyocyte glucose uptake and cardiac function concomitant with the activation of PPARγ and AMPK^[277]^. AMPK can also enhance the expression and translocation of GLUT4 and glucose uptake, which is particularly beneficial in the context of diabetic cardiomyopathy^[312]^. Resveratrol is a naturally occurring polyphenolic stilbene that has been shown to increase mitochondrial biogenesis through both AMPK and NO-dependent mechanisms through activation of PGC-1α, NRFs, and TFAM. Importantly, several studies have reported improved mitochondrial biogenesis and cardiac functional parameters with resveratrol administration during hypertension-mediated HF in both humans and animals without a measurable reduction in blood pressure, suggesting direct effects on the heart^[313–316]^. Various direct activators of AMPK have also been developed and tested; however, the development of AMPK activators is complicated by variable expression levels and differential effects of various subunits and isoforms^[317]^. For example, several groups have reported that amino acid substitution within the C-terminal side of the γ2 subunit of AMPK leads to the development of aberrant conduction systems and severe cardiac hypertrophy^[318–320]^, although it has been suggested that this hypertrophy phenotype may be attributable to increased carbohydrate storage as opposed to myocyte cytoskeletal growth^[320,321]^. Interestingly, it has also been observed that systemic pan-activation of AMPK, for example, by MK-8722, induced as opposed to ameliorated cardiac hypertrophy^[322]^.

Another promising avenue for increasing mitochondrial biogenesis is the soluble guanylyl cyclase/cyclic guanosine monophosphate (sGC/cGMP) pathway. Nitric oxide synthesized in the vasculature binds to sGC in vessel smooth muscle, catalyzing the conversion of guanosine triphosphate to cGMP. cGMP clearance is regulated by hydrolyzing phosphodiesterases, including PDE5A, which is known to be expressed in cardiomyocytes^[323]^. cGMP and its effector kinase, PKG, have been shown to regulate cardiac structure and function *via* regulation of calcium flux, phosphorylation of contractile proteins, and several other mechanisms [Figure 5]^[324]^. It has also been suggested that this pathway stimulates mitochondrial biogenesis *via* PGC-1α and inhibits mitochondrially mediated cell death^[325–327]^. sGC stimulators have been previously used to treat pulmonary hypertension and have recently emerged as potential therapeutics for heart failure^[305,306]^. Recent clinical trials with the sGC stimulator vericiguat showed some promise by significantly reducing high-risk HFrEF patient hospitalization^[328]^ and improving patient quality of life^[329]^, nevertheless, no recent trials have shown benefits of sGC/cGMP stimulation in patients with HFpEF^[306,330]^. As PDE5 expression is upregulated in hypertrophic and failing hearts, leading to decreased cGMP levels, inhibition of PDE5 represents an attractive therapeutic target, and it has been shown that the PDE5 inhibitor, sildenafil, ameliorates PO-induced cardiac hypertrophic remodeling in mice *via* deactivation of PKG and inhibition of the L-type Ca^2+^ channel^[331]^.

Increasing evidence from animal models suggests that targeted inhibition of ROS within mitochondria, rather than globally, may be cardioprotective, as it was shown in the GISSI-Prevenzione trial that chronic supplementation with the global antioxidant α-tocopherol resulted in an *increased* risk of the development of HF in postinfarction patients^[332]^. Thus, agents that specifically target mitochondrial ROS should be prioritized. MitoQ is a lipophilic quinol that accumulates in the mitochondrial matrix by association with triphenylphosphonium^[333]^. Mitochondrial ROS are scavenged by oxidizing MitoQ to its quinone form, which is subsequently recycled back into the quinol form by ETC complex II^[334]^. A recent study in rats with PO-induced HF showed that MitoQ reduced H_2_O_2_ levels and improved mitochondrial respiration [Figure 5]^[335]^. While MitoQ is widely considered a safe, orally bioavailable treatment with minimal off-target effects, its efficacy could be limited in severe HF where mitochondrial membrane potential is reduced, since its uptake is directly driven by membrane potential.

Szeto-Schiller peptides are small antioxidant molecules that rapidly accumulate in mitochondria, given their high affinity for cardiolipin. Elamipretide (Bendavia) is a Szeto-Schiller peptide that appears to exert cardioprotective effects by reducing mitochondrial ROS and limiting maladaptive cardiac remodeling^[336,337]^. A 2016 study showed that long-term elamipretide treatment improved systolic function, increased cardiac mitochondrial respiratory capacity, ATP production, and restored mitochondrial membrane potential, and reduced circulating inflammatory markers in dogs with advanced heart failure, and another study showed improved *ex vivo* function in mitochondria from failing human hearts^[338,339]^ [Figure 5]. However, elamipretide has failed to show promising results in later-stage clinical trials in patients with various heart failure phenotypes (NCT02814097, NCT02914665, NCT02788747). Genetic overexpression of MnSOD and mitochondrial catalase have been shown to limit mitochondrial ROS, reduce cardiac hypertrophy, and improve in both type I and type II diabetic mice. Thus, it is unsurprising that SOD-mimetics such as mitoTEMPO, a mitochondrially targeted free radical scavenger, have also been successful in improving cardiac outcomes in various models of heart failure^[340–342]^. Another promising antioxidant compound is N-acetylcysteine, which is an FDA-approved drug that is known to mediate cellular redox imbalance by increasing intracellular concentrations of glutathione^[343]^, which was shown to normalize oxidative stress levels in diabetic rats and prevent the development of diabetic cardiomyopathy after 5 weeks of supplementation^[344,345]^.

Sodium-glucose cotransporters are secondary active symporters of sodium and glucose, with the SGLT2 isoform expressed in the proximal tubule of renal nephrons^[346]^. Inhibition of SGLT2 reduces renal glucose reabsorption and results in glucose concentration-dependent glucosuria, ameliorating hyperglycemia without the risk of inducing hypoglycemia^[347]^. SGLT2 inhibitors have proven to significantly reduce cardiovascular mortality and heart failure hospitalizations in at least three large clinical trials of type II diabetic patients^[348–351]^. Beyond their glucose-lowering properties, SGLT2 inhibitors may provide additional cardioprotective effects by modulating sodium homeostasis. Regulation of sodium kinetics is crucial for maintaining cardiomyocyte mitochondrial redox balance and excitation-contraction coupling. Thus, these inhibitors might improve mitochondrial energetics and oxidative defense by attenuating intracellular sodium overload. It has also been suggested that SGLT2 inhibitors may improve mitochondrial dynamics through activation of AMPK, which alters DRP1 phosphorylation leading to suppression of mitochondrial fission^[352,353]^.

Remarkably, additional cardioprotective benefits are increasingly believed to be conferred independently of glucose and sodium lowering, and it has been shown that SGLT2 inhibition with empagliflozin ameliorates adverse cardiac remodeling and enhances cardiac bioenergetic function in non-diabetic animal models of HF. In particular, in non-diabetic rats post-myocardial infarction, empagliflozin attenuated cardiomyocyte hypertrophy, diminished interstitial fibrosis, reduced myocardial oxidative stress, reduced mtDNA damage, and stimulated mitochondrial biogenesis [Figure 5]^[354–356]^. These changes were associated with improvements in the pattern of glucose and fatty acid uptake and oxidation. Additionally, SGLT2 inhibitors have been widely shown to increase circulating ketone body levels, which has been postulated to occur *via* lowering of portal insulin-to-glucagon ratio, causing lipolysis and increased FFA delivery to the liver. Concurrently, SGLT2 inhibition has been associated with increased myocardial expression of the ketone body transporter and enzymes, indicating increased utilization of ketone bodies as an alternate efficient fuel source, resulting in significantly increased cardiac ATP production^[354]^.

Finally, as the importance of mitochondrial dynamics and mitophagy is increasingly recognized, the field should continue to develop therapeutic strategies that target or mimic mitophagy in the heart^[357]^. Exercise and caloric restriction are both potent nonpharmacologic stimulators of mitophagy *via* activation of the AMPK-ULK1 pathway, facilitating the clearance of damaged mitochondria and limiting the associated inflammation^[358–360]^. It follows that fasting-memetic drugs may similarly upregulate mitophagy, and indeed both the allosteric mTOR inhibitor rapamycin and the aliphatic polyamine spermidine have been shown to stimulate mitophagy in cardiomyocytes in mice, restoring the proteome to a more youth composition and improving cardiac outcomes, as well as prolonging life [Figure 5]^[357,361,362]^. Given the encouraging developments in therapies and drugs targeting autophagy and mitophagy in the heart, this area likely offers further therapeutic promise, and future research efforts should consider leveraging this axis to improve cardiac function and outcomes in the context of aging, heart disease, and heart failure.

## CONCLUSION

The heart is the most energy-consuming organ in the body, and cardiac contractile machinery requires a constant supply of ATP to maintain systemic circulation. Thus, the heart displays remarkable metabolic flexibility in adapting to increased demands. However, chronically dysregulated systemic metabolism, persistent PO, and the natural decline in bioenergetic function and molecular milieu associated with aging set in motion a bi-directional cascade of mitochondrial dysfunction and structural remodeling in the heart. The mechanisms by which mitochondrial dysfunction occurs and potentiates cardiac structural changes have been widely studied and were reviewed in Sections (“TRIGGERS OF CARDIAC MITOCHONDRIAL DYSFUNCTION” and “MITOCHONDRIAL DYSFUNCTION DRIVES CARDIAC REMODELING”). Relatively fewer studies have explored the inverse relationship: the effects of myocardial structural remodeling on mitochondrial milieu and bioenergetic demand (Section “CARDIAC REMODELING ALTERS MITOCHONDRIAL BIOENERGETICS”). The bidirectionality of structural and energetic remodeling cascades may prove particularly important during the transition and decompensation stages of heart failure, when the pathological mechanical and biochemical environment overwhelms disrupted energy systems beyond their compensatory capacity. Thus, future studies of the mechanistic progression to heart failure should consider altered mitochondrial function as both a cause and consequence of structural cardiac remodeling. We then discuss the encouraging development of therapies and drugs targeting mitochondrial function to improve cardiac outcomes in the context of aging, heart disease, and heart failure.

## Data Availability

Not applicable.
